# Prehospital time and mortality in pediatric trauma

**DOI:** 10.1007/s00383-024-05742-9

**Published:** 2024-06-20

**Authors:** Olivia Nieto Rickenbach, Joshua Aldridge, Dmitry Tumin, Erika Greene, Matthew Ledoux, Shannon Longshore

**Affiliations:** 1 Brody School of Medicine at East, Carolina University, 600 Moye Blvd, Greenville, NC 27858 USA; 2 ECU Health Medical Center, Greenville, NC USA; 3 Department of Surgery, Brody School of Medicine at East, Carolina University, Greenville, NC USA; 4 Department of Pediatrics, Brody School of Medicine at East, Carolina University, Greenville, NC USA

**Keywords:** Trauma, Golden hour, Mortality, Pediatrics

## Abstract

**Purpose:**

The “Golden Hour” of transportation to a hospital has long been accepted as a central principal of trauma care. However, this has not been studied in pediatric populations. We assessed for non-linearity of the relationship between prehospital time and mortality in pediatric trauma patients, redefining the threshold at which reducing this time led to more favorable outcomes.

**Methods:**

We performed an analysis of the 2017–2018 American College of Surgeons Trauma Quality Improvement Program, including trauma patients age < 18 years. We examined the association between prehospital time and odds of in-hospital mortality using linear, polynomial, and restricted cubic spline (RCS) models, ultimately selecting the non-linear RCS model as the best fit.

**Results:**

60,670 patients were included in the study, of whom 1525 died and 3074 experienced complications. Prolonged prehospital time was associated with lower mortality and fewer complications. Both models demonstrated that mortality risk was lowest at 45–60 min, after which time was no longer associated with reduced probability of mortality.

**Conclusions:**

The demonstration of a non-linear relationship between pre-hospital time and patient mortality is a novel finding. We highlight the need to improve prehospital treatment and access to pediatric trauma centers while aiming for hospital transportation within 45 min.

**Supplementary Information:**

The online version contains supplementary material available at 10.1007/s00383-024-05742-9.

## Introduction

The “Golden Hour” has long been a tenet of surgical and trauma care, indicating the critical period of emergency care during which trauma patients’ lives are hanging in the balance. There has been considerable effort to develop hospital and trauma systems that minimize time to definitive treatment for the injured patient, ensuring that there is appropriate triage and rapid transportation available to the appropriate level of trauma center [1]. These efforts reflect the finding that most trauma-related deaths occur early (within the first several hours) after a traumatic injury, and that even basic first aid delivered during this time may be crucial in saving patients’ lives [2–4]. Multiple studies in adults have validated the relationship between reduced pre-hospital time and improved patient outcomes, with one such study demonstrating a threefold increase in the odds of survival when transport time is less than 60 min [5–9]. In rural areas, minimizing pre-hospital time requires extensive investment in the air and ground transport, including contracting to third-party emergency medical transport companies when hospital-operated and public transport services are insufficient.

The challenges with completing patient transport within the “Golden Hour” may be particularly pertinent in pediatric trauma patients, as fewer numbers of pediatric trauma centers may leave a traumatically injured pediatric patient hundreds of miles from the nearest Level I Pediatric center and at increased risk of short-term mortality [10]. Furthermore, the providers at smaller, referring centers and local or volunteer Emergency Medical Services (EMS) providers may not have experience in caring for pediatric trauma patients [11, 12]. With the “Golden Hour” concept focusing on improving trauma patient survival, it is important to determine in what way this threshold of 60 min influences mortality and other clinical outcomes. Evidence in adult studies is mixed, with some demonstrating a linear association with transport time and increased mortality, while others suggest that prolonged pre-hospital time functions as a “trial of life” for trauma patients [13–18]. Other adult studies demonstrate an inverse relationship between transport time and patient outcomes, which may be function of a mature trauma system that appropriately triages patients [19].

Apart from the mixed evidence in adult patients, there is limited evidence regarding prehospital transport times and their association with mortality in pediatric trauma patients. If this association is linear (shorter prehospital times consistently reducing mortality risk to the same degree), then further reduction of prehospital time below the “Golden Hour” threshold may be warranted. Alternately, if this association is not linear, then identifying the threshold at which longer prehospital time becomes more strongly associated with mortality can help refine the concept of the “Golden Hour,” either by increasing or decreasing this conventional target. In this study, we used data from a multicenter trauma registry to test the hypothesis that mortality risk in pediatric trauma varies non-linearly with prehospital time (i.e., that each additional minute of prehospital time does not necessarily imply the same change in mortality risk). If a non-linear relationship between mortality and prehospital time were supported by the data, our secondary aim was to describe the range of prehospital times within which delayed arrival at the hospital was most strongly associated with survival.

## Methods

This analysis was not considered human subjects research by the Institutional Review Board, because it used deidentified data. We analyzed the 2017–2018 American College of Surgeons Trauma Quality Improvement Program (TQIP), a national database consisting of over 700 participating trauma centers and over a million admissions [20]. Of these admissions, pediatric patients (age 0–17 years) with blunt or penetrating trauma were included in this study if they were transported by EMS directly from the scene of the injury. We excluded patients with unknown prehospital time, prehospital times below the 1st or above the 99th percentiles (< 15 min or > 133 min), and missing data on study outcomes or other variables.

The primary outcome was in-hospital mortality. Secondary outcomes included hospital length of stay (LOS), need for intensive care unit (ICU) admission, and any hospital complications (as defined in the TQIP data, outlined in Appendix Table 1). Prehospital time was defined as a continuous variable representing the time elapsed from initial dispatch of EMS to patient arrival at the hospital. This total prehospital time included EMS response time, EMS time on the scene, and transport time back to the hospital [15]. Demographic covariates included patient age, sex, race and ethnicity, and insurance status. Clinical covariates include mechanism of injury (blunt vs. penetrating), transportation mode (ground vs. air), injury severity score (ISS), and pediatric trauma center verification level (Level I, Level II, or none).

Bivariate logistic regression was used to characterize the shape of the association between prehospital time and odds of in-hospital mortality. We examined a linear model (where the odds of mortality increase by the same multiplier for each minute increase in prehospital time), and two types of nonlinear models, using either a polynomial specification or a restricted cubic spline (RCS) specification of prehospital time. In the polynomial model, higher powers of prehospital time (e.g., quadratic transformation of prehospital time, cubic transformation, and so on) were added to the regression equation until the Wald test demonstrated no further improvement in model fit (e.g., when comparing cubic to quadratic transformation). In the RCS model, prehospital time was divided at 5 knots based on previously recommended percentiles [21]. The final model specification was chosen based on visual inspection of predicted probabilities and a comparison of receiver operating characteristics (ROC) curves [22].

Patient characteristics were summarized for the overall sample using means with standard deviations (SD) and counts with percentages. Using 60 min as an a priori cutoff for prehospital time, we compared patient characteristics and outcomes between patients with < 60 and ≥ 60 min prehospital time via Chi-square tests and independent *t* tests. We then fit a separate multivariable regression model for each outcome, including prehospital time as a continuous variable and all covariates described above. Logistic regression was used for binary outcomes, and linear regression was used for continuous outcomes. For each outcome, we plotted the predicted probability (or predicted values) with a 95% confidence interval (CI) over the range of prehospital times in our analysis, with categorical covariates set to their reference category and continuous covariates set to the sample mean. In a post hoc analysis, we re-fitted each regression model of study outcomes to the subsample of patients with penetrating trauma, who were determined to have shorter prehospital times than patients experiencing blunt trauma. Data analysis was completed in Stata/SE 16.1 (College Station, TX: StataCorp, LP). *P* < 0.05 was considered statistically significant.

## Results

We identified 76,449 pediatric patients arriving from the scene after suffering blunt or penetrating trauma. We excluded 12,997 cases missing data on prehospital time, 1,088 cases with extremely short (< 15 min) or extremely long (> 133 min) prehospital time, 1,344 cases missing data on study outcomes, and 350 cases missing data on covariates. The final sample included 60,670 cases (65% male/35% female; age 12 ± 5 years), of whom 3% (*N* = 1525) died, 5% experienced in-hospital complications (*N* = 3074), and 21% (*N* = 12,807) were admitted to the ICU. Average LOS was 4 ± 7 days, and average prehospital time was 48 ± 21 min. Prehospital time was < 60 min for 75% of patients.

On univariate logistic regression analysis (predicted values plotted in Appendix Fig. 1), all specifications of prehospital time indicated decreasing risk of mortality as prehospital time increased. Both the cubic polynomial and the restricted cubic spline models suggested that mortality risk was lowest at prehospital times of 45–60 min. The restricted cubic spline model was selected for as the preferred specification of prehospital time for further analyses, based on having the largest area under the ROC curve (C-statistic of 0.657).

Study variables are summarized for the overall sample and compared by prehospital time (< 60 vs. ≥ 60 min) in Table 1. Patients with prolonged prehospital time (≥ 60 min) were more likely to be female, non-Hispanic White, and covered by private insurance. In addition, prolonged prehospital time was associated with blunt trauma, air as compared to ground transportation, and admission to a level I pediatric trauma center. Considering study outcomes, the bivariate analysis in Table 1 found that prolonged prehospital time was associated with lower mortality (2 vs. 3%), fewer in-hospital complications (4 vs. 5%), lower likelihood of admission to the ICU (20 vs. 21%), but higher total LOS (mean of 4.0 vs. 3.7 days).

**Table 1:** Study variables in the overall sample and stratified by prehospital time (*N* = 60,670)

Variable	Mean (SD) or *N* (%)			*P*
	All patients (*N* = 60,670)	Prehospital time < 60 min (*N* = 45,619)	Prehospital time ≥ 60 min (*N* = 15,051)	
Mortality	1525 (3%)	1282 (3%)	243 (2%)	< 0.001
LOS (days)	3.8 (7.1)	3.7 (7.5)	4.0 (5.7)	< 0.001
ICU admission	12,807 (21%)	9755 (21%)	3052 (20%)	0.004
In-hospital complications	3074 (5%)	2433 (5%)	641 (4%)	< 0.001
Age (years)	11.5 (5.0)	11.5 (5.1)	11.5 (4.8)	0.242
Sex				
Male	39,690 (65%)	30,352 (67%)	9338 (62%)	< 0.001
Female	20,980 (35%)	15,267 (33%)	5713 (38%)	
Race/ethnicity				
Non-Hispanic White	30,114 (50%)	20,399 (45%)	9715 (65%)	< 0.001
Non-Hispanic Black	14,153 (23%)	12,044 (26%)	2109 (14%)	
Hispanic or Latino	11,290 (19%)	9163 (20%)	2127 (14%)	
None of the above	5113 (8%)	4013 (9%)	1100 (7%)	
Insurance coverage				
Private	26,993 (45%)	19,650 (43%)	7343 (49%)	< 0.001
Public	25,585 (42%)	19,940 (44%)	5645 (38%)	
Other	3286 (5%)	2321 (5%)	965 (6%)	
None	4806 (8%)	3708 (8%)	1098 (7%)	
Mechanism of injury				
Blunt trauma	53,562 (88%)	39,307 (86%)	14,255 (95%)	< 0.001
Penetrating trauma	7108 (12%)	6312 (14%)	796 (5%)	
Transport mode				
Ground	53,820 (89%)	42,731 (94%)	11,089 (74%)	< 0.001
Air	6850 (11%)	2888 (6%)	3962 (26%)	
ISS	8.6 (9.2)	8.6 (9.4)	8.8 (8.6)	< 0.001
Pediatric trauma center verification level				
None	38,940 (64%)	29,971 (66%)	8969 (60%)	< 0.001
Level I	14,623 (24%)	10,289 (23%)	4334 (29%)	
Level II	7107 (12%)	5359 (12%)	1748 (12%)	

*ICU* Intensive care unit; *ISS* injury severity score; *LOS* length of stay; *SD* standard deviation

**Fig. 1:**
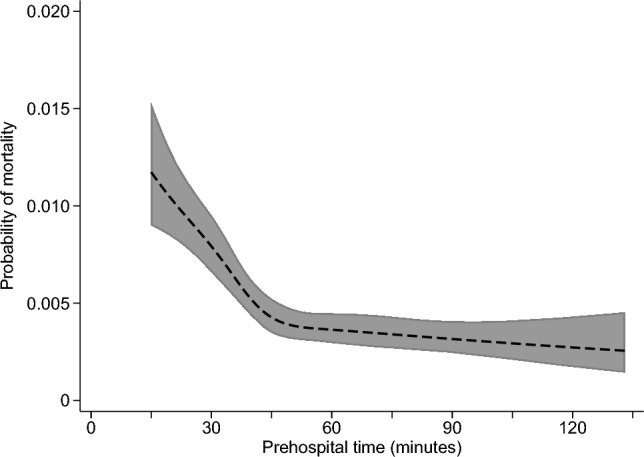
Predicted probability (with 95% confidence interval) of mortality according to prehospital time, based on multivariable logistic regression

**Fig. 2:**
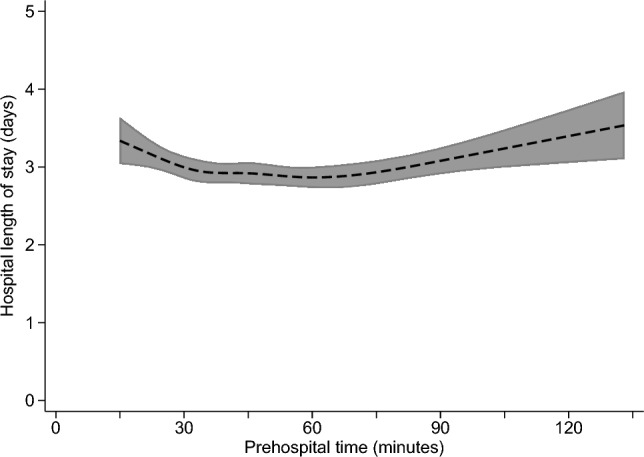
Predicted hospital length of stay (with 95% confidence interval) according to prehospital time, based on multivariable linear regression

**Fig. 3:**
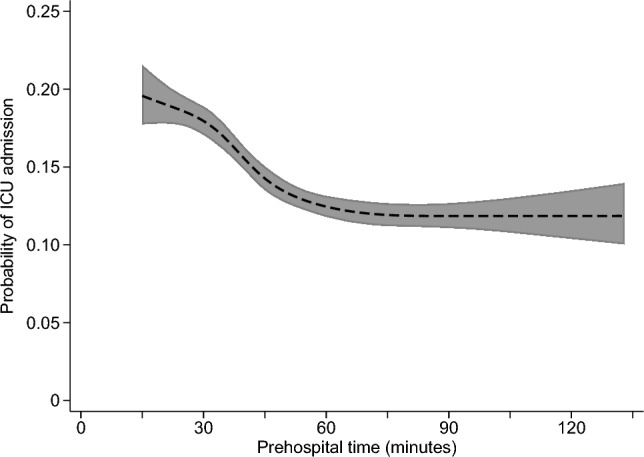
Predicted probability (with 95% confidence interval) of intensive care unit (ICU) admission according to prehospital time, based on multivariable logistic regression

**Fig. 4:**
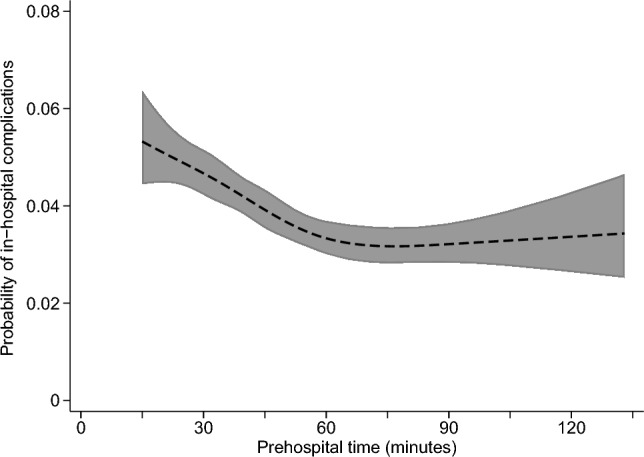
Predicted probability (with 95% confidence interval) of in-hospital complications according to prehospital time, based on multivariable logistic regression

The adjusted relationship of prehospital time with each study outcome is summarized in Figs. 1, 2, 3 and 4, with full results from each multivariable model shown in Appendix Tables 2–5. The predicted probability of mortality was highest for the shortest prehospital times in the sample, declining steadily until about 45 min of prehospital time, when its association with prehospital time became much weaker (Fig. 1). A similar pattern was observed for the association between prehospital time and ICU admission (Fig. 3) or in-hospital complications (Fig. 4), each of which were most likely for patients with the shortest prehospital times, and became less likely as prehospital time approached 45–60 min. Considering total LOS, prehospital time was not evidently associated with this outcome at times < 75 min, with longer prehospital times beyond this threshold predicting slightly longer hospital LOS (Fig. 2).

Results were similar in a post hoc subgroup analysis of patients with penetrating trauma, with probability of mortality being highest for patients with the shortest prehospital times, and declining steadily until approximately 45 min of prehospital time (Appendix Fig. 2). In this subgroup, LOS and probability of ICU admission were highest among patients with the shortest prehospital time, and declined until approximately 30 min of prehospital time (Appendix Figs. 3, 4). There was no evident association between prehospital time and probability of in-hospital complications in the subset of patients experiencing penetrating trauma (Appendix Fig. 5).

In the primary analysis, other factors associated with increased odds of mortality, ICU admission, and in-hospital complications on multivariable analysis included penetrating mechanism of injury, air rather than ground transport, and higher injury severity. Lack of health insurance coverage was associated with greater odds of mortality (odds ratio [OR]: 3.31; 95% confidence interval [CI] 2.77, 3.95; *P* < 0.001), but decreased odds of ICU admission (OR: 0.66; 95% CI 0.60, 0.72; *P* < 0.001), and decreased odds of experiencing in-hospital complications (OR: 0.65; 95% CI 0.55, 0.76; *P* < 0.001). Patient and injury characteristics associated with prolonged LOS included air transport, penetrating mechanism of injury, admission to a level I pediatric trauma center, and African American race or Hispanic ethnicity.

## Discussion

Our study demonstrates a non-linear relationship between prehospital time and in-hospital outcomes among pediatric trauma patients surviving until hospital arrival. Previous work has centered on the concept of the “Golden Hour” window to hospital arrival, and highlighted the importance of minimizing prehospital time to improve survival [1, 5]. However, our analysis found that the risk of in-hospital mortality declined with longer prehospital time linearly up to around 30–45 min, which represented the prehospital time with the lowest risk for in-hospital mortality. After this threshold, prehospital time was no longer associated with increased risk of mortality. High mortality rates among patients arriving in < 30 min likely reflect appropriate triaging of the most critically injured patients. Nonetheless, it is important to recognize that these data provide no evidence for increasing survival after hospital admission associated with reducing prehospital time.

Considering the high in-hospital mortality rates seen among patients arriving within 30 min, our results suggest that critical pediatric traumas may require a threshold lower than the traditional 60 min to hospital arrival (“Golden Hour”) to optimize survival. On the other hand, among less critically injured pediatric trauma patients with prehospital times greater than 45 min, outcomes were similar regardless of prehospital times. For this group of patients, since prehospital time was not associated with in-hospital outcomes, focusing on other strategies such as transport to a verified pediatric trauma center may improve outcomes despite further prolongation of prehospital time. This strategy is consistent with prior results documenting that treatment of pediatric trauma patients at a level I pediatric trauma center is associated with a survival benefit for these patients [23]. However, we acknowledge that there are too few such centers in the United States to accommodate the volume of pediatric traumas, as compared to adult trauma centers. For example, only 72% of pediatric trauma patients in the United States have access to a verified pediatric trauma center accessible within 60 min by the air or ground transportation [24], and only 53% of pediatric trauma patients in the 2017–2018 TQIP registry received care in a pediatric-verified trauma center [25].

In addition to the previously described “Golden Hour,” the concept of the “Platinum 10 min” describes the goal time on the scene of injury for pre-hospital providers prior to transport. The need for patient extrication, presence of multiple patients, or concerns for scene safety in the setting of firearm injuries can significantly extend this time. It is common for EMS providers to exceed this 10-min goal, with only one-third of patients meeting this transportation window in one study [13, 26]. EMS providers must arrive on the scene with the equipment, personnel, and skills necessary to rapidly stabilize and transport critically injured patients [16]. The experience of EMS providers with pediatric trauma patients and the procedural difference in this specific population may also contribute to a lower success rate with prehospital interventions. There is evidence that while prehospital interventions do not delay care [27], the number of intubations performed, and their success rates are lower in pediatric trauma patients than in adults [28–30]. The effect of procedural unfamiliarity may be increased in rural areas, where pediatric trauma patients are treated less frequently.

The demonstration of a non-linear relationship between pre-hospital time and patient mortality is a novel finding in pediatric trauma. The “Golden Hour” concept has mixed validity in adult studies, with recent research failing to identify a relationship between pre-hospital time and mortality in this patient population [17, 31]. Paradoxically, longer transport time may be a marker of stability, as more critically injured patients could be transported more quickly to the nearest hospital. Our results agree with Chen et al. suggesting that pre-hospital time is an important metric for the trauma population [15]. In that study, there was an inflection point within the first 30 min at which reductions in prehospital time could influence patient mortality. There is evidence that helicopter EMS transport can improve survival within the 30-min post-trauma window [13], particularly in blunt trauma. However, this increased survival is attributed to improved access to verified trauma centers for patients requiring a higher level of care [32]. Our results suggest that trauma systems should consider a more aggressive strategy in transportation of pediatric trauma patients, especially for those in rural areas that are less likely to have access to trauma system resources. Availability of helicopter EMS services across these rural regions, not limited to helicopters that are based within hospitals, could facilitate faster patient transport.

There are multiple other interventions trauma systems could employ to meet a guideline for patient transport within 30 min. For many trauma systems, this guideline would require increased helicopter EMS usage. This transportation method is expensive to maintain, costly to patients and insurers, and not without risks [33]. This method is also the most severely impacted by weather. To help improve prehospital treatment and meet a 30-min guideline, trauma systems may focus on EMS training for pediatric care and procedures. This would require increased outreach by pediatric trained providers, more frequent in-service training, and collaboration across EMS agencies. However, this may result in fewer private EMS companies available for transportation of patients, which could increase prehospital time due to delays in care. Therefore, trauma systems may choose to focus on improving the availability of pediatric care throughout their system or region, reducing the need for transport to a distant center.

Increasing the number of verified pediatric trauma centers, particularly Level 2 or Level 3, within a region would reduce the prehospital time in pediatric trauma patients. There are significant financial, regulatory, and personnel burdens to the verification process, which may preclude the feasibility of this intervention in many hospital systems. Finally, systems could identify a scoring or triage system to better identify those patients at highest risk of mortality in the field. As suggested by Chen et al. [15], those patients with prehospital hypotension, Glasgow Coma Scale score less than 8, and thoracoabdominal firearm injuries are more likely to have time sensitive injuries and are most likely to benefit from transportation times of less than 30 min. These criteria may be a starting point for determining transportation methods and urgency.

We acknowledge that interventions to improve the care of injured patients can be costly, and may not address the issue of injury prevention. Especially in rural areas and areas with limited health system resources, it may not be realistic to target a reduction in prehospital time as the main way to improve survivability of pediatric injuries, and these interventions would need to be combined with interventions to increase the effectiveness of prehospital interventions (e.g., improving EMS training for pediatric trauma and airways), and interventions focusing on prevention of common causes of fatal injury, such as non-accidental trauma, motor vehicle crashes, and firearm-related injuries [34].

While the TQIP database is a robust, nationwide database, this study faced several limitations. Despite the large sample size, many patients were excluded from the study due to missing data, which may have impacted our findings. Likewise, the use of deidentified data limits our ability to identify specific patient, injury, hospital factors that affected prehospital time and patient outcomes, transportation method selection, as well as whether patients were transported to a verified pediatric trauma center while bypassing the closest hospital. Furthermore, the primary outcome of in-hospital mortality does not account for mortality prior to arrival to definitive hospital care. The choice of statistical approach for modeling non-linearity also may have impacted our results. Though the chosen restricted cubic spline model demonstrated the highest C-statistic, the cubic polynomial model had a similar fit, and utilization of this model could have altered our conclusions.

## Conclusions

In summary, our study demonstrates a novel result in the prehospital care of pediatric trauma patients. We demonstrate a linear association between increasing pre-hospital time and decreasing in-hospital mortality in the 30–45 min post-trauma, suggesting that an alternative guideline to the traditional “Golden Hour” may be appropriate for pediatric trauma. Trauma systems should continue to focus on rapid triage and transport of severely injured children to reduce prehospital time and mortality. Strategies for achieving these goals may include increased helicopter usage, pediatric specific training, transportation scoring systems, or verification of additional pediatric trauma centers across the United States.

## Supplementary Information

Below is the link to the electronic supplementary material.

Supplementary file1 Predicted probability of mortality, based on unadjusted logistic regression models including prehospital time using a linear, polynomial (cubic), or restricted cubic spline specification (PDF 30 KB)

Supplementary file2 Predicted probability (with 95% confidence interval) of mortality according to prehospital time, based on multivariable logistic regression, among patients sustaining penetrating trauma (N=7,108) (PDF 31 KB)

Supplementary file3 Predicted hospital length of stay (with 95% confidence interval) according to prehospital time, based on multivariable linear regression (N=7,108) (PDF 31 KB)

Supplementary file4 Predicted probability (with 95% confidence interval) of intensive care unit (ICU) admission according to prehospital time, based on multivariable logistic regression (N=7,108) (PDF 31 KB)

Supplementary file5 Predicted probability (with 95% confidence interval) of in-hospital complications according to prehospital time, based on multivariable logistic regression (N=7,108) (PDF 31 KB)

Supplementary file6 (PDF 65 KB)

Supplementary file7 (PDF 78 KB)

Supplementary file8 (PDF 78 KB)

Supplementary file9 (PDF 78 KB)

Supplementary file10 (PDF 123 KB)

## Data Availability

No datasets were generated or analysed during the current study.
