# Supplementary figures and images for: Prehospital time and mortality in pediatric trauma
Source: Pediatr Surg Int. 2024 Jun 20;40(1):159. doi: 10.1007/s00383-024-05742-9 (PMC11190012; doi:10.1007/s00383-024-05742-9)

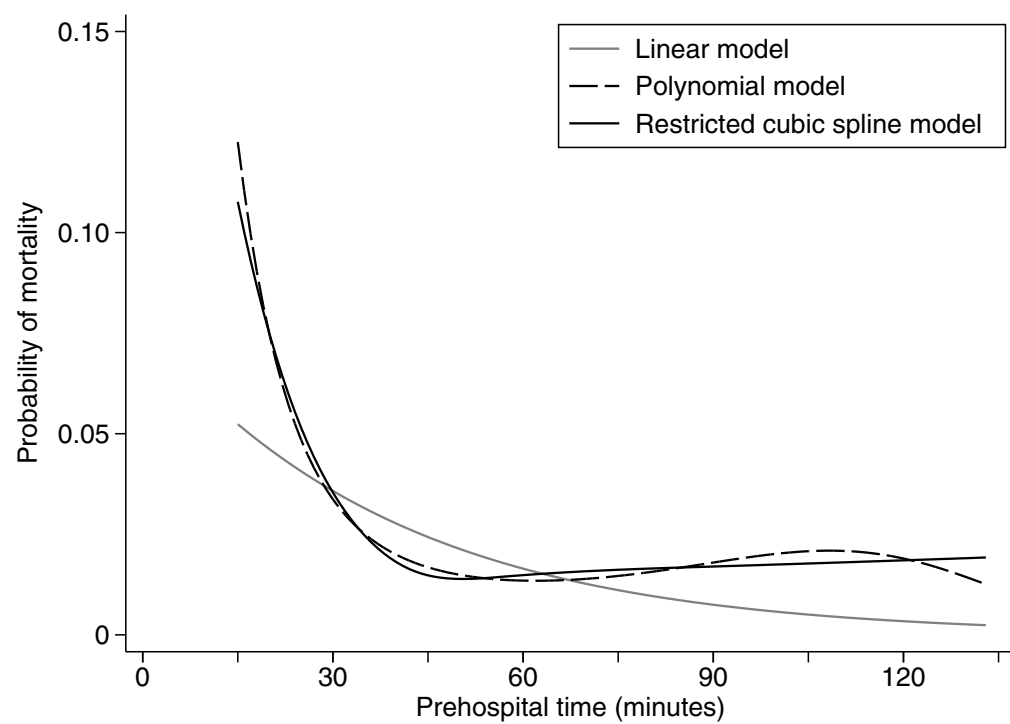

Supplement: Supplementary file 1 — Supplementary file1 Predicted probability of mortality, based on unadjusted logistic regression models including prehospital time using a linear, polynomial (cubic), or restricted cubic spline specification (PDF 30 KB) [file 383_2024_5742_MOESM1_ESM.pdf]

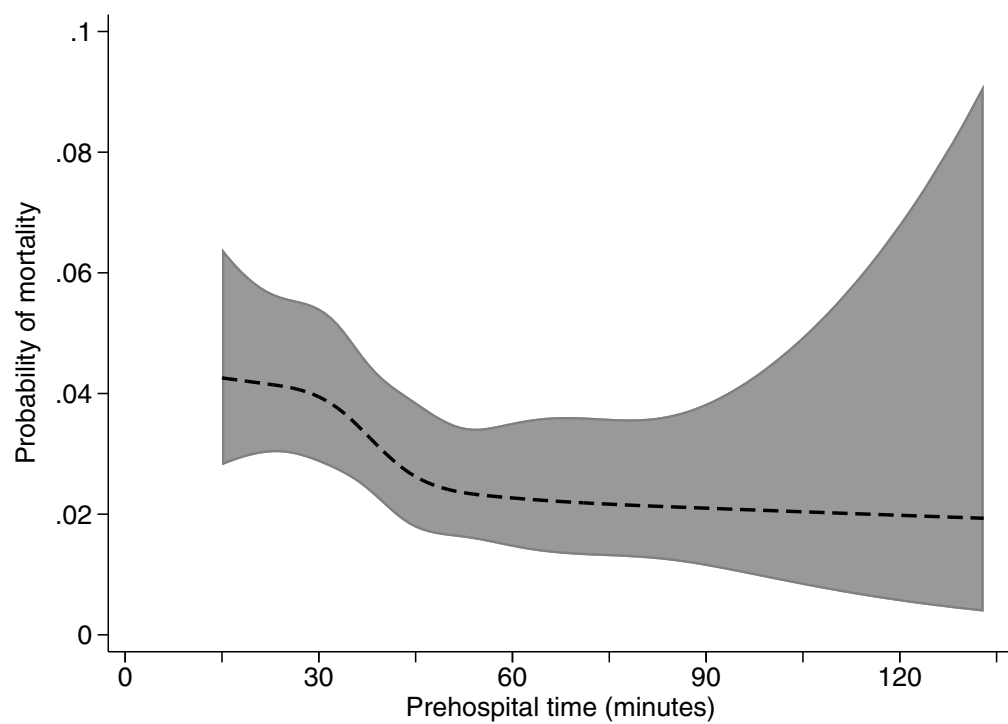

Supplement: Supplementary file 2 — Supplementary file2 Predicted probability (with 95% confidence interval) of mortality according to prehospital time, based on multivariable logistic regression, among patients sustaining penetrating trauma (N=7,108) (PDF 31 KB) [file 383_2024_5742_MOESM2_ESM.pdf]

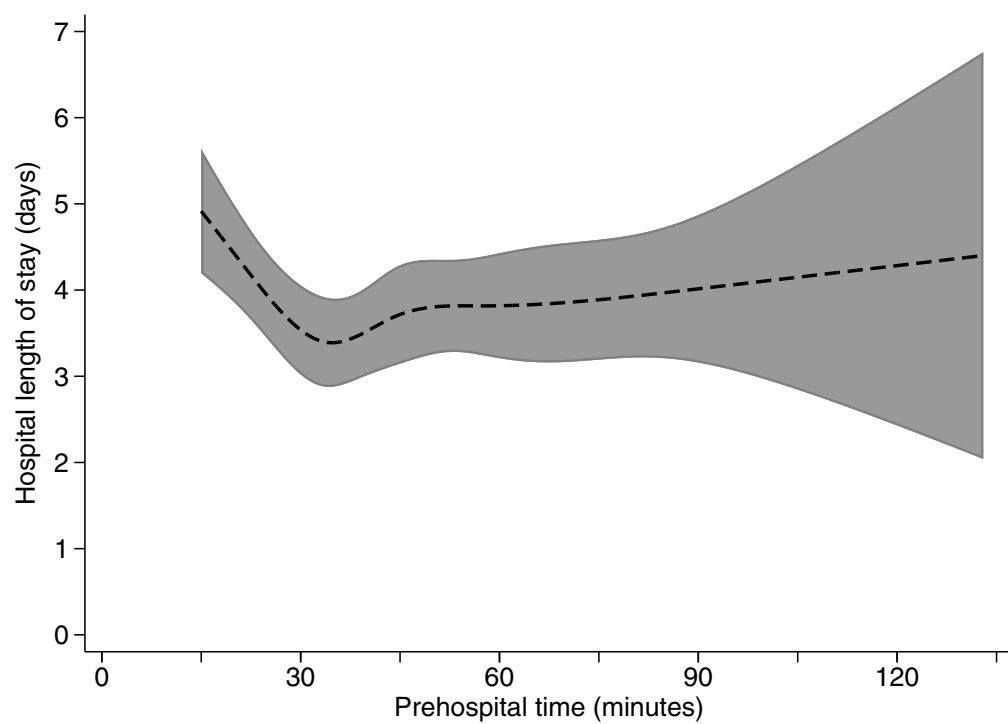

Supplement: Supplementary file 3 — Supplementary file3 Predicted hospital length of stay (with 95% confidence interval) according to prehospital time, based on multivariable linear regression (N=7,108) (PDF 31 KB) [file 383_2024_5742_MOESM3_ESM.pdf]

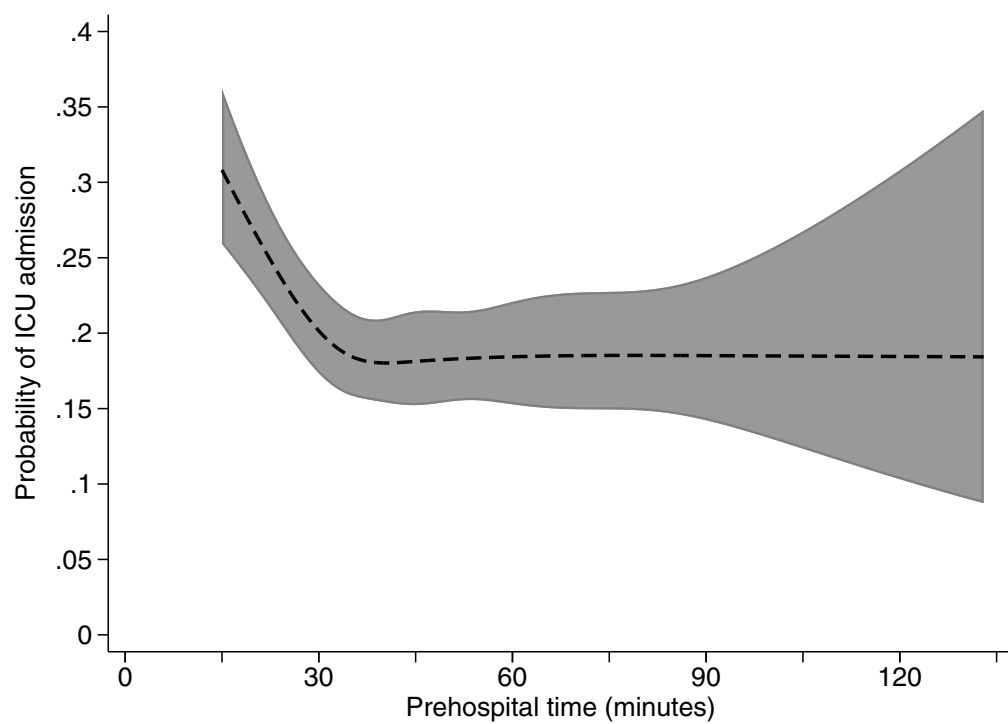

Supplement: Supplementary file 4 — Supplementary file4 Predicted probability (with 95% confidence interval) of intensive care unit (ICU) admission according to prehospital time, based on multivariable logistic regression (N=7,108) (PDF 31 KB) [file 383_2024_5742_MOESM4_ESM.pdf]

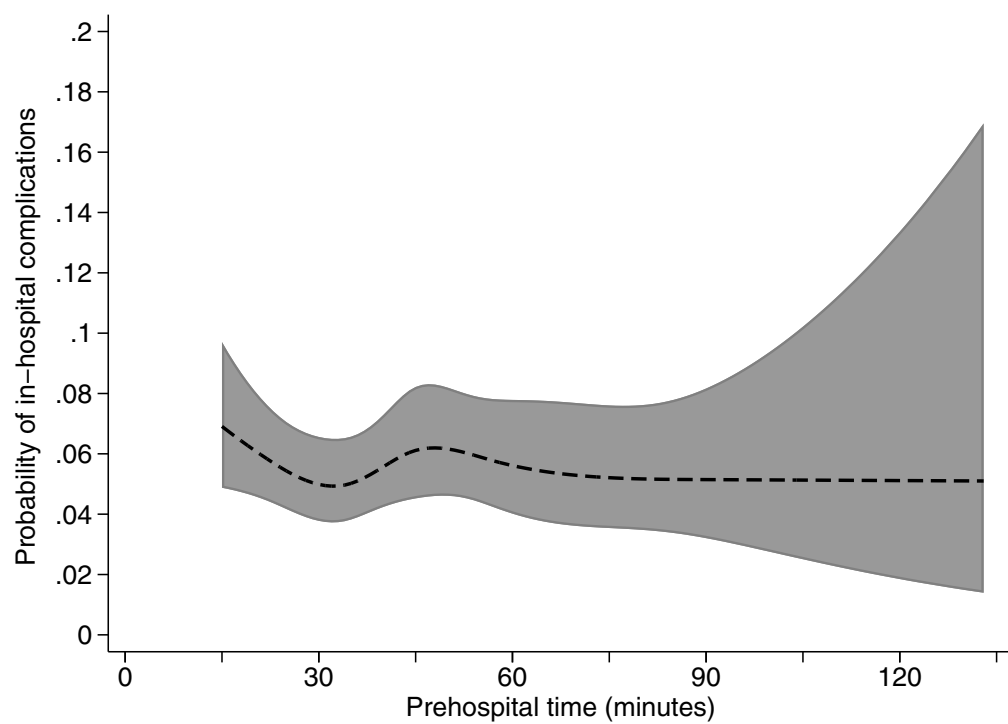

Supplement: Supplementary file 5 — Supplementary file5 Predicted probability (with 95% confidence interval) of in-hospital complications according to prehospital time, based on multivariable logistic regression (N=7,108) (PDF 31 KB) [file 383_2024_5742_MOESM5_ESM.pdf]
