# Supplementary material for: Prehospital time and mortality in pediatric trauma
Source: Pediatr Surg Int. 2024 Jun 20;40(1):159. doi: 10.1007/s00383-024-05742-9 (PMC11190012; doi:10.1007/s00383-024-05742-9)
Supplement: Supplementary file 6 — Supplementary file6 (PDF 65 KB) [file 383_2024_5742_MOESM6_ESM.pdf]

Prehospital time and mortality in pediatric trauma

Pediatric Surgery International

Corresponding author: Olivia Nieto Rickenbach, BA. Brody School of Medicine at East Carolina University, 600 Moye Blvd, Greenville NC 27858 USA. Email: orickenbach@gmail.com.

Appendix Table 1. In-hospital complications as defined in TQIP data

**Cardiovascular**	**Respiratory**	**Infectious**
Cardiac arrest w/ cardiopulmonary resuscitation (CPR)	Acute respiratory distress syndrome (ARDS)	Deep surgical site infection
		Organ/space surgical site infection
Deep vein thrombosis (DVT)	Pulmonary embolism (PE)	
		Osteomyelitis
Myocardial infarction (MI)	Unplanned intubation	
		Severe sepsis
Acute kidney injury (AKI)		
		Catheter-associated UTI
Stroke/Cerebrovascular accident (CVA)		
		Central line associated bloodstream infection
		Ventilator-associated pneumonia
		Superficial incisional surgical site infection
