# Supplementary material for: Prehospital time and mortality in pediatric trauma
Source: Pediatr Surg Int. 2024 Jun 20;40(1):159. doi: 10.1007/s00383-024-05742-9 (PMC11190012; doi:10.1007/s00383-024-05742-9)
Supplement: Supplementary file 7 — Supplementary file7 (PDF 78 KB) [file 383_2024_5742_MOESM7_ESM.pdf]

**Prehospital time and mortality in pediatric trauma**

Pediatric Surgery International

Corresponding author: Olivia Nieto Rickenbach, BA. Brody School of Medicine at East Carolina University, 600 Moye Blvd, Greenville NC 27858 USA. Email: orickenbach@gmail.com.

**Appendix Table 2.** Multivariable logistic regression of mortality (N=60,670).

| Variable | OR | 95% CI | P |
|---|---|---|---|
| Prehospital time (minutes)[a] | | | |
| All times | 0.98 | 0.96, 0.99 | 0.009 |
| Times >22 minutes | 0.84 | 0.65, 1.08 | 0.171 |
| Times >34 minutes | 2.09 | 0.89, 4.94 | 0.092 |
| Times >44 minutes | 0.44 | 0.18, 1.09 | 0.075 |
| Age (years) | 0.97 | 0.96, 0.99 | <0.001 |
| Sex | | | |
| Male | Ref. | | |
| Female | 1.10 | 0.93, 1.21 | 0.365 |
| Race/ethnicity | | | |
| Non-Hispanic White | Ref. | | |
| Non-Hispanic Black | 0.99 | 0.84, 1.16 | 0.885 |
| Hispanic or Latino | 0.92 | 0.77, 1.10 | 0.348 |
| None of the above | 0.94 | 0.74, 1.19 | 0.600 |
| Insurance coverage | | | |
| Private | Ref. | | |
| Public | 0.96 | 0.83, 1.11 | 0.589 |
| Other | 1.53 | 1.18, 1.20 | 0.002 |
| None | 3.31 | 2.77, 3.95 | <0.001 |
| Mechanism of injury | | | |

| | | | |
|---|---|---|---|
| Blunt trauma 1 | Ref. | | |
| Penetrating trauma 2 | 5.4 | 4.70, 6.25 | <0.001 |
| Transport mode | | | |
| Ground 2 | Ref. | | |
| Air 1 | 2.24 | 1.86, 2.70 | <0.001 |
| ISS | 1.12 | 1.11, 1.12 | <0.001 |
| Pediatric trauma center verification level | | | |
| None | Ref. | | |
| Level I | 0.96 | 0.82, 1.12 | 0.595 |
| Level II | 1.08 | 0.89, 1.31 | 0.441 |

CI, confidence interval; ICU, intensive care unit; ISS, injury severity score; OR, odds ratio

[a] In the restricted cubic spline model, all times contribute to prediction of the outcome via the first term, times above the 22 minute cutoff contribute to prediction of the outcome via the first and second terms, times above the 33 minute cutoff contribute to prediction of the outcome via the first three terms, and times above the 44 minute cutoff contribute to prediction of the outcome via all four terms.
