# Supplementary material for: Prehospital time and mortality in pediatric trauma
Source: Pediatr Surg Int. 2024 Jun 20;40(1):159. doi: 10.1007/s00383-024-05742-9 (PMC11190012; doi:10.1007/s00383-024-05742-9)
Supplement: Supplementary file 8 — Supplementary file8 (PDF 78 KB) [file 383_2024_5742_MOESM8_ESM.pdf]

Prehospital time and mortality in pediatric trauma

Pediatric Surgery International

Corresponding author: Olivia Nieto Rickenbach, BA. Brody School of Medicine at East Carolina University, 600 Moye Blvd, Greenville NC 27858 USA. Email: orickenbach@gmail.com.

Appendix Table 3. Multivariable linear regression of hospital length of stay in days (N=60,670).

Variable	Coefficient	95% CI	P
Prehospital time (minutes)[a]			
All times	-0.02	-0.05, -0.00	0.033
Times >22 minutes	0.16	-0.09, 0.41	0.217
Times >34 minutes	-0.42	-1.20, 0.36	0.290
Times >44 minutes	0.37	-0.40, 1.14	0.348
Age (years)	0.06	0.05, 0.07	<0.001
Sex			
Male			
Female	0.07	-0.04, 0.18	0.206
Race/ethnicity			
Non-Hispanic White			
Non-Hispanic Black	0.42	0.28, 0.57	<0.001
Hispanic or Latino	0.40	0.25, 0.55	<0.001
None of the above	0.38	0.18, 0.57	<0.001
Insurance coverage			
Private			
Public	0.38	0.26, 0.50	<0.001
Other	0.42	0.18, 0.66	0.001
None	-0.84	-1.04, -0.64	<0.001

Mechanism of injury			
Blunt trauma			
Penetrating trauma	0.69	0.52, 0.86	<0.001
Transport mode			
Ground			
Air	1.44	1.26, 1.62	<0.001
ISS	0.26	0.26, 0.27	<0.001
Pediatric trauma center verification level			
None			
Level I	0.74	0.61, 0.87	<0.001
Level II	0.47	0.30, 0.63	<0.001

CI, confidence interval; ICU, intensive care unit; ISS, injury severity score

[a] In the restricted cubic spline model, all times contribute to prediction of the outcome via the first term, times above the 22 minute cutoff contribute to prediction of the outcome via the first and second terms, times above the 33 minute cutoff contribute to prediction of the outcome via the first three terms, and times above the 44 minute cutoff contribute to prediction of the outcome via all four terms.
