# Supplementary material for: Prehospital time and mortality in pediatric trauma
Source: Pediatr Surg Int. 2024 Jun 20;40(1):159. doi: 10.1007/s00383-024-05742-9 (PMC11190012; doi:10.1007/s00383-024-05742-9)
Supplement: Supplementary file 9 — Supplementary file9 (PDF 78 KB) [file 383_2024_5742_MOESM9_ESM.pdf]

Prehospital time and mortality in pediatric trauma

Pediatric Surgery International

Corresponding author: Olivia Nieto Rickenbach, BA. Brody School of Medicine at East Carolina University, 600 Moye Blvd, Greenville NC 27858 USA. Email: orickenbach@gmail.com.

Appendix Table 4. Multivariable logistic regression of intensive care unit admission (N=60,670).

Variable	OR	95% CI	P
Prehospital time (minutes)[a]			
All times	0.99	0.98, 1.00	0.168
Times >22 minutes	0.90	0.81, 0.10	0.041
Times >34 minutes	1.40	1.01, 1.94	0.044
Times >44 minutes	0.76	0.54, 1.05	0.094
Age (years)	1.00	0.99, 1.00	0.391
Sex			
Male	Ref.		
Female	0.98	0.93, 1.02	0.321
Race/ethnicity			
Non-Hispanic White	Ref.		
Non-Hispanic Black	1.06	1.00, 1.13	0.046
Hispanic or Latino	1.24	1.16, 1.32	<0.001
None of the above	1.21	1.11, 1.31	<0.001
Insurance coverage			
Private	Ref.		
Public	1.02	0.97, 1.08	0.376
Other	0.89	0.80, 0.99	0.034
None	0.66	0.60, 0.72	<0.001

Mechanism of injury			
Blunt trauma	Ref.		
Penetrating trauma	1.21	1.12, 1.3	<0.001
Transport mode			
Ground	Ref.		
Air	2.50	2.34, 2.68	<0.001
ISS	1.13	1.12, 1.13	<0.001
Pediatric trauma center verification level			
None	Ref.		
Level I	1.05	0.99, 1.10	0.100
Level II	1.47	1.37, 1.57	<0.001

CI, confidence interval; ICU, intensive care unit; ISS, injury severity score; OR, odds ratio

[a] In the restricted cubic spline model, all times contribute to prediction of the outcome via the first term, times above the 22 minute cutoff contribute to prediction of the outcome via the first and second terms, times above the 33 minute cutoff contribute to prediction of the outcome via the first three terms, and times above the 44 minute cutoff contribute to prediction of the outcome via all four terms.
